# Hippocampal connectivity predicting recognition and categorization abilities

**DOI:** 10.3389/fcogn.2026.1765217

**Published:** 2026-03-31

**Authors:** Kyla Brannigan, Lea Frank, Dagmar Zeithamova

**Affiliations:** Brain and Memory Lab, Department of Psychology, University of Oregon, Eugene, OR, United States

**Keywords:** anterior hippocampus, face blend, generalization, integration, posterior hippocampus, separation, specificity

## Abstract

**Introduction:**

Flexible use of memory involves both the ability to form detailed memories of individual experiences (specificity) and to generalize across related experiences (generalization). Memory specificity and generalization have been attributed to distinct neocortical regions, such as ventrolateral prefrontal cortex (PFC) and ventromedial PFC respectively. The hippocampus has been traditionally associated with memory specificity, but more recent work highlights additional role in generalization. Here, we tested the hypothesis that the hippocampus supports both memory specificity and generalization, but through interactions with distinct cortical regions.

**Methods:**

Fifty-two adults learned to categorize blended face stimuli, enabling both extraction of category structure (generalization) and encoding of item-specific features (specificity). Background functional connectivity was measured using fMRI during passive viewing of the same faces before and after learning.

**Results:**

Participants showed robust category learning, above-chance recognition of studied faces from similar lures, and successful category generalization to novel category members. Recognition and categorization performance were not highly correlated, suggesting distinct processes supporting each memory function. In the brain, we found distinct connectivity profiles of anterior hippocampus, presumed to preferentially contribute to generalization, and posterior hippocampus, presumed to preferentially contribute to specificity. Learning led to increased anterior hippocampal connectivity with default mode regions including ventromedial PFC, and posterior hippocampal connectivity with visual cortex. Increased anterior hippocampal connectivity with ventromedial PFC, somatomotor cortex, and visual cortex predicted better category generalization, whereas increased posterior hippocampal connectivity with ventrolateral PFC predicted more accurate face recognition. Exploratory analyses revealed widespread learning-related changes in cortico-cortical interactions, with changes in connectivity among visual, somatomotor, and default mode networks predicting categorization.

**Discussion:**

Together, these findings support the notion that the hippocampus supports both memory specificity and generalization through interactions with distinct cortical networks. These results advance mechanistic accounts of how the hippocampus and cortex coordinate to balance competing memory demands.

## 1 Introduction

The ability to remember the details of our experiences (specificity) and the ability to generalize across experiences to create new knowledge (generalization) are two key functions of healthy memory. It is well established that memory specificity relies on the hippocampus (Eichenbaum, 2000; Scoville and Milner, 1957; Tulving and Markowitsch, 1998). More recent work has shown that the hippocampus may also play a role in supporting memory generalization (Preston et al., 2004; Shohamy and Wagner, 2008; Zeithamova et al., 2008, 2012). Multiple recent models propose that hippocampus may support multiple memory functions by representing events at multiple levels of specificity, although the nature of the division of labor within the hippocampus varies across models (Berens and Bird, 2017; Poppenk et al., 2013; Schapiro et al., 2017).

One theory suggests that information is represented in different levels of detail along the long-axis of the hippocampus, with representations in the anterior hippocampus being more coarse-grained and general, and representations in the posterior hippocampus being more fine-grained and specific (Poppenk et al., 2013). This hypothesis builds on research in animal models showing that receptive fields in hippocampal place cells increase in size from dorsal to ventral hippocampus, suggesting that information is represented across gradually larger spatial scales (Kjelstrup et al., 2008). This gradient has been extended to humans as well. For example, Brunec et al. (2018) showed that voxel dynamics are more highly correlated in the anterior than posterior hippocampus and that anterior hippocampus has higher temporal autocorrelation than posterior hippocampus. These findings taken together corroborate the idea that representations in the anterior hippocampus are larger in scale while posterior hippocampus may represent more detailed information. Anterior-posterior hippocampal dissociations have also been demonstrated for other aspects of memory, such as cognitive processes or stimulus type (Grady, 2020), suggesting that functional dissociations between anterior and posterior hippocampus may be a consistent property of the hippocampus spanning multiple domains.

Along with functional differences within the hippocampus, regions in the cortex also differentially contribute to memory specificity and memory generalization. Although some regions, such as the angular gyrus, have been implicated in both memory specificity (Kuhl and Chun, 2014; Xiao et al., 2017) and memory generalization (Morton et al., 2020), other regions have been strongly associated with one memory function only. Previous studies have shown that a network of regions including lateral prefrontal and lateral parietal cortices support memory specificity by resolving interference between similar items in memory (Badre and Wagner, 2005; Bowman and Dennis, 2016; Jonker et al., 2018; Kuhl et al., 2007; Kuhl and Chun, 2014; Uncapher and Wagner, 2009). In contrast, a network of regions including medial prefrontal and lateral temporal cortices appear primarily involved in memory generalization (Bowman and Zeithamova, 2025). For example, the ventromedial PFC is involved in integrating common information from previous experiences (Schlichting et al., 2015), encoding new information with respect to preexisting knowledge (Van Kesteren et al., 2013), and applying derived categorical information to new examples (Bowman and Zeithamova, 2018). Lateral temporal regions, such as the middle temporal gyrus, are thought to represent conceptual knowledge (Bowman and Zeithamova, 2018; Davis and Poldrack, 2014) and gist information (Dennis et al., 2007; Turney and Dennis, 2017). Thus, memory specificity and generalization may be supported by dissociable neural systems.

Despite evidence that specific and generalized memories are represented both within the hippocampus and the cortex, less is known about how the hippocampus and cortex interact to support these functions. Recent studies have started to investigate how hippocampal coupling with distributed cortical systems memory specificity and generalization functions. For instance, hippocampal connectivity with the inferior frontal gyrus is associated with the ability to discriminate memories upon retrieval (Bowman and Dennis, 2016; Manelis et al., 2013), lending support to the idea that hippocampal connections with putative memory specificity cortical regions support specificity abilities. In contrast, hippocampal-ventromedial PFC connectivity has been shown to correlate with ability to integrate memories during encoding (Bowman and Zeithamova, 2018; Frank et al., 2019; Schlichting et al., 2015; Zeithamova et al., 2012). This suggests that hippocampal connections with putative memory generalization cortical regions relate to generalization abilities. The anterior and posterior hippocampus have distinct connectivity profiles (Chase et al., 2015; Dalton et al., 2022; Tang et al., 2020), which may support their complementary memory functions. For example, one study showed that the anterior hippocampus primarily interacts with the network of regions implicated in memory generalization, including ventromedial PFC and anterior lateral temporal cortex while posterior hippocampus is more strongly connected with a network of regions that are implicated in memory specificity, including lateral prefrontal and lateral parietal cortices (Frank et al., 2019). Thus, the hippocampus may support different memory functions by interacting with distinct regions.

Investigating learning-related changes in inter-regional interactions has been a fruitful tool for understanding memory functions and representations (Ranganath and Ritchey, 2012; Schlichting et al., 2015; Tambini and Davachi, 2013). For example, work on post-encoding awake reactivation highlights that hippocampal–cortical coupling and coordinated fluctuations after learning can shape later memory and bias cognition (Tambini and Davachi, 2019). Relatedly, whole-brain analyses demonstrate that recent learning can produce measurable functional reconfiguration of brain circuits, emphasizing that memory-related change can be expressed at the level of network organization rather than single connections or regions (Passiatore et al., 2021). Consistent with this systems perspective, learning can also rapidly establish enduring memory-related representations outside the hippocampus, including in posterior parietal cortex (Brodt et al., 2018). Thus, post-learning changes in hippocampal functional coupling and network-level reconfiguration may help elucidate how different brain systems contribute to the formation of specific and generalized representations and memory functions that rely on them.

While prior studies provided initial evidence that the hippocampus may interact with distinct cortical regions to support memory specificity and generalization, the studies differed in tasks, stimuli, timing, and other parameters, making it difficult to uniquely attribute the regional differences to distinct memory functions. In the current study, we utilized a single learning paradigm that allowed us to assess both the representations of individual experiences and generalization across experiences for the same stimuli, in the same participants (Ashby and Zeithamova, 2022; Bowman et al., 2022). Participants underwent a category learning task with blended faces while we assessed background hippocampal-cortical connectivity before and after the learning task. Participants subsequently completed a recognition task, putatively relying on specific memories, and a categorization task, putatively relying on memory generalization. Our goals were to characterize hippocampal-cortical connectivity along the long-axis of the hippocampus, how the connectivity changes in response to experience, and how the changes predict individual differences in memory for specific details of the experience and the ability to generalize. All data associated with this project are publicly available at https://osf.io/5h8b7.

## 2 Materials and methods

### 2.1 Participants

Sixty-two healthy individuals participated in this study. Participants were eligible for the MRI if they were right-handed, were native English speakers, had no psychiatric or neurological illness, and were not currently taking medications known to affect brain function. Before the experiment, all participants gave informed consent in accordance with a protocol approved by the Institutional Review Board of the University of Oregon. Data from 10 participants were excluded for falling asleep during the task (six participants), for program error (two participants), and for non-compliance with study procedures (two participants). Data from the remaining 52 participants are included in all analyses (18 males, 33 females, 1 non-binary person; ages 18–28; mean age = 19.8 years).

### 2.2 Experimental design

#### 2.2.1 Overview of tasks

**Figure 1:**
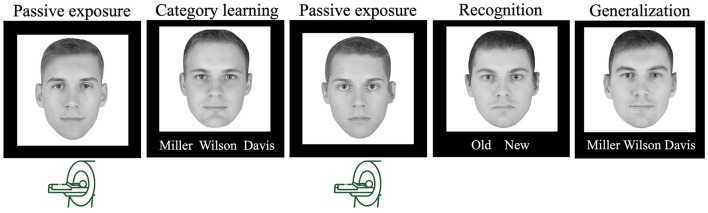
Experimental design. The study consisted of five phases: a pre-learning passive exposure phase (scanned), a category training phase (completed during anatomical scan), a post-learning passive exposure phase (scanned), a recognition memory task (behavioral, outside scanner), and a category generalization task (behavioral, outside scanner). Only the passive exposure task was scanned. The recognition memory and category generalization behavioral tasks were completed outside of the scanner.

The study procedure, shown in Figure 1, consisted of five experimental phases: a pre-learning passive exposure phase (scanned), a category training phase (completed during anatomical scan), a post-learning passive exposure phase (scanned), a recognition phase, and a categorization phase. A resting-state scan was also completed but not considered in this paper.

##### 2.2.1.1 Pre-training passive exposure

First, participants passively viewed images of 9 training faces that were presented 4 times each over 2 runs, for a total of 36 trials. Participants were instructed to pay attention to each face but did not have to make any responses while viewing them. Functional MRI data from the pre-learning passive exposure phase were used to calculate background connectivity measures reported in this manuscript.

##### 2.2.1.2 Category learning task

After the passive exposure phase, participants entered the category training phase. Here, participants were shown the faces they had seen in the passive exposure paradigm, but now they had to learn to sort each face into its respective family. The family structure is shown in Figure 1. After being shown each face, participants had to respond with a button-press which family the face belonged to. They would receive immediate feedback whether they chose the correct family. The 9 training faces were presented 16 times each over 2 blocks, for a total of 288 trials. Timing was self-paced. The anatomical scan was collected during the category learning task to utilize the time that participant was spending in the scanner.

##### 2.2.1.3 Post-training passive exposure

Following the category training task, participants completed a second passive exposure phase, identical to the pre-training one. Participants paid attention to each face they were shown and were not asked to make any responses. Functional MRI data from the post-learning passive exposure phase were used to assess how hippocampal-cortical connectivity changed after learning.

##### 2.2.1.4 Recognition test

After the second round of passive exposure, participants completed a self-paced recognition task outside of the scanner. Participants were shown intermixed training and novel test faces, making old/new judgements for each face with a key press. While a combination of 9 old and 42 new faces creates an unbalanced old/new ratio, we have previously demonstrated that participants can successfully differentiate old from new stimuli well above chance (Ashby et al., 2020). We also verified here that the corrected hit rate score (hit rate minus false alarm rate) served as a reliable recognition score suitable for individual difference analysis. Performance on the recognition task served as our measure of memory specificity.

##### 2.2.1.5 Categorization test

Following the recognition task, participants completed a self-paced categorization test. In this task, participants were shown all 51 test faces and were asked to sort these faces into their respective families based on the category structure they had previously learned. This time they received no feedback on their responses. Performance on the categorization test served as our measure of memory generalization. While recognition and categorization are not process-pure measures and may contain contributions from both specific and generalized representations, we have previously demonstrated that they are not highly correlated in this task (Bowman et al., 2020; Bowman and Zeithamova, 2018). Furthermore, brain-behavior analyses utilized multiple regression with both categorization and recognition in the same regression model to further focus on unique variance captured by each task.

#### 2.2.2 Stimuli

**Figure 2:**
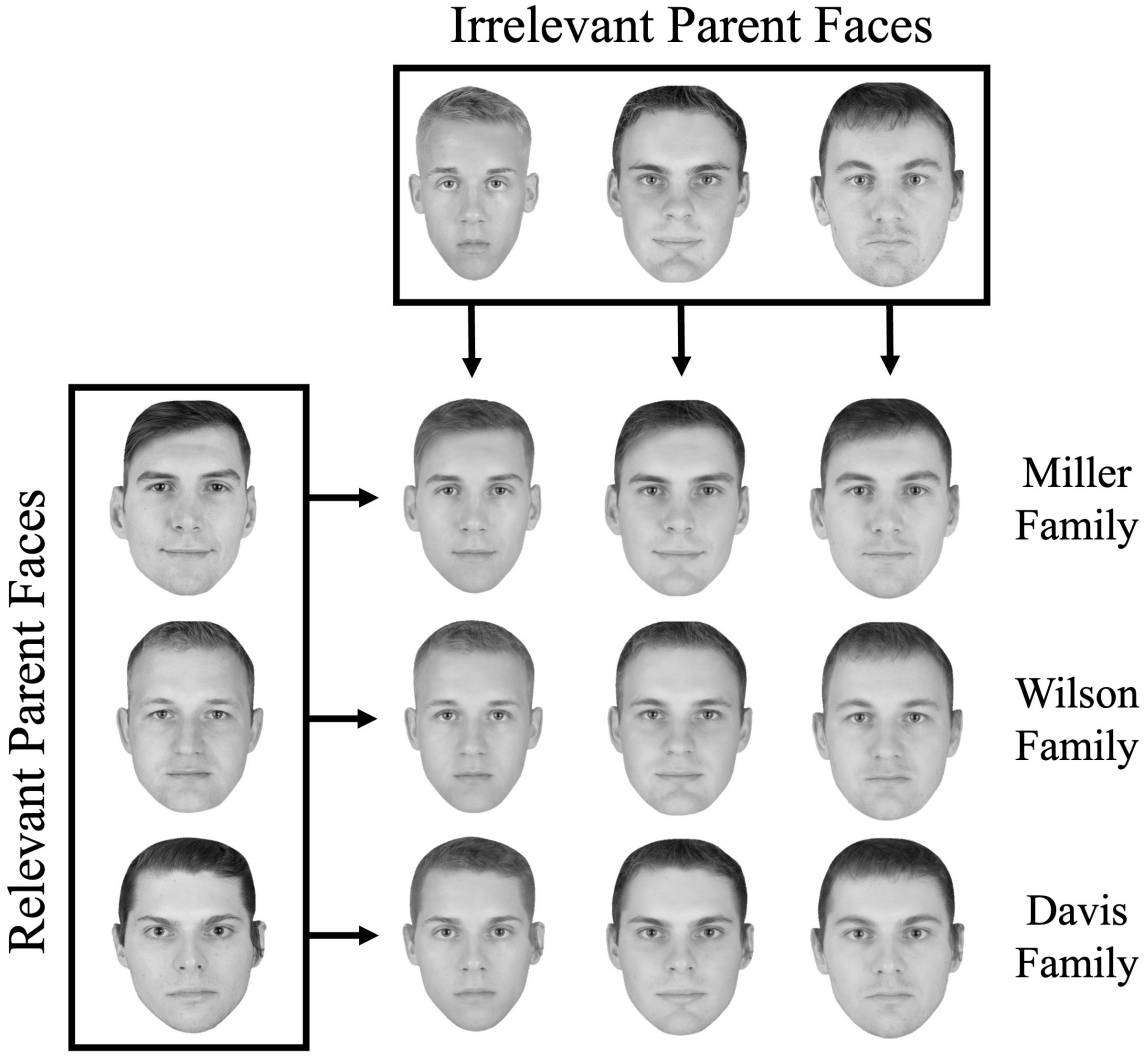
Category structure. Each category is created by blending one category-relevant parent face with three category-irrelevant parent faces, generating three members of each family.

Stimuli for this study were images of white male faces with a neutral expression. The face-blend stimuli, category structure, and paradigm were adapted from previous studies by Ashby, Zeithamova, and colleagues (Ashby et al., 2020; Ashby and Zeithamova, 2022; Bowman et al., 2022). Faces needed to be all of one gender and all of one race, because otherwise pre-existing categories would interact and/or interfere with new category learning. Each stimulus was a 50/50 blend of one *category relevant parent face* and one *category irrelevant parent face* (Figure 2). The relevant parent face was associated with a last name that indicated which category (or family) the blended faces belonged to. Irrelevant parent faces contained no information regarding category membership, but contributed to the face blend's unique appearance.

There were a total of 20 parent faces. To create a set of training faces, each participant was randomly assigned three parent faces to serve as category relevant and three parent faces as category irrelevant training faces. Each category's relevant parent face was blended with each of the three irrelevant parent faces to generate nine unique training faces, three from each category (Figure 1). The set of nine training faces was used in the category learning task and in the passive exposure phases. The remaining 14 parent faces were used to create additional novel faces for recognition and categorization tests. Each relevant parent face was blended with the 14 never-seen irrelevant parent faces to generate 14 new members of each category. The recognition and categorization stimulus sets consisted of the 9 training faces and 42 novel test faces, for a total of 51 test faces. To control for item effects, we used data from prior studies with these stimuli and limited the pool of faces from which relevant and irrelevant parents were randomly drawn to 10 faces with intermediate similarity to other faces (not too distinctive, not too average), as determined by prior similarity ratings (Ashby et al., 2020). Because the selection of relevant and irrelevant faces, and the assignment of relevant faces to family names were randomized across participants, any observed brain-behavior correlations would be unlikely to reflect item effects.

### 2.3 Behavioral statistical analyses

The category generalization and recognition tasks were used to operationalize memory generalization and memory specificity, respectively. We reasoned that the category generalization performance may index participants' ability to extract commonalities and form generalizable category representations during the category learning task. We reasoned that recognition performance may index participants' ability to form specific representations of individual faces during the category learning task that can help individuate them from other similar faces during recognition.

#### 2.3.1 Categorization performance

Categorization performance was measured as the accuracy for categorizing testing faces during the category generalization task. A one-sample *t*-test compared categorization performance to chance (33.3% for three categories).

#### 2.3.2 Recognition performance

Recognition performance was measured using a corrected hit rate (hit rate–false alarm rate) from the recognition task to determine how well participants were able to differentiate the training faces from new similar faces. A one-sample *t*-test comparing corrected hit rate to zero was used to assess whether recognition was above chance.

### 2.4 FMRI acquisition and preprocessing

#### 2.4.1 Acquisition

Scans were completed on a 3T Siemens MAGNETOM Skyra scanner equipped with a 32-channel head coil at the University of Oregon Lewis Center for Neuroimaging. Foam padding was used to minimize head motion. The scanning session consisted of a localizer scan, one 8-min resting-state scan (not considered in the current paper), four 3.67-min runs of a passive exposure task, and two anatomical scans. Functional data were acquired using a multiband gradient-echo pulse sequence (TR, 2,000 ms; TE, 25 ms; flip angle, 90°; matrix size, 104 × 104; 72 contiguous slices oriented 15° off the anterior commissure–posterior commissure line to reduce prefrontal signal dropout; interleaved acquisition; FOV, 208 mm; voxel size, 2.0 × 2.0 × 2.0 mm; GRAPPA factor, 2; multiband acceleration factor, 3). A standard T1-weighted (T1w) MPRAGE anatomical image (TR, 2,500 ms; TE, 3.43 ms; TI, 1,100 ms; flip angle, 7°; matrix size, 256 × 256; 176 contiguous sagittal slices; FOV, 256 mm; slice thickness, 1 mm; voxel size, 1.0 × 1.0 × 1.0 mm; GRAPPA factor, 2) was collected between the second and third functional runs. Participants completed the category learning task during this anatomical scan. The final scan was a custom anatomical T2 coronal image (TR, 13,520 ms; TE, 88 ms; flip angle, 150°; matrix size, 512 × 512; 65 contiguous slices oriented perpendicularly to the main axis of the hippocampus; interleaved acquisition; FOV, 220 mm; voxel size, 0.4 × 0.4 × 2 mm; GRAPPA factor, 2). The current paper only focuses on the pre- and post- category learning passive exposure scans to test how hippocampal connectivity changes in response to learning and how pre-to-post changes predict memory for specific stimulus information (recognition) and generalization ability (categorization).

#### 2.4.2 Preprocessing using fMRIPrep

Results included in this manuscript come from preprocessing performed using FMRIPREP version 20.2.0 (Esteban et al., 2019, RRID:SCR_016216), a Nipype (Gorgolewski et al., 2011, 2017, RRID:SCR_002502) based tool. Each T1w volume was corrected for INU (intensity non-uniformity) using N4BiasFieldCorrection v2.1.0 (Tustison et al., 2010) and skull-stripped using antsBrainExtraction.sh v2.1.0 (using the OASIS template). Brain surfaces were reconstructed using recon-all from FreeSurfer v6.0.1 (Dale et al., 1999, RRID:SCR_001847) and the brain mask estimated previously was refined with a custom variation of the method to reconcile ANTs-derived and FreeSurfer-derived segmentations of the cortical gray matter of Mindboggle (Klein et al., 2017, RRID:SCR_002438). Spatial normalization to the ICBM 152 Nonlinear Asymmetrical template version 2009c (Fonov et al., 2009, RRID:SCR_008796) was performed through nonlinear registration with the antsRegistration tool of ANTs v2.1.0 (Avants et al., 2008, RRID:SCR_004757), using brain-extracted versions of both T1w volume and template. Brain tissue segmentation of cerebrospinal fluid (CSF), white matter (WM) and gray matter (GM) was performed on the brain-extracted T1w using fast (FSL v5.0.9, Behzadi et al., 2007, RRID:SCR_002823).

Functional data were slice time corrected using 3dTshift from AFNI v16.2.07 (Cox, 1996, RRID:SCR_005927) and motion corrected using mcflirt (FSL v5.0.9, Jenkinson et al., 2002). This was followed by co-registration to the corresponding T1w using boundary-based registration (Greve and Fischl, 2009) with six degrees of freedom, using bbregister (FreeSurfer v6.0.1). Motion correcting transformations, BOLD-to-T1w transformation and T1w-to-template (MNI) warp were concatenated and applied in a single step using antsApplyTransforms (ANTs v2.1.0) using Lanczos interpolation.

Physiological noise regressors were extracted applying CompCor (Behzadi et al., 2007). Principal components were estimated for the two CompCor variants: temporal (tCompCor) and anatomical (aCompCor). A mask to exclude signal with cortical origin was obtained by eroding the brain mask, ensuring it only contained subcortical structures. Six tCompCor components were then calculated including only the top 5% variable voxels within that subcortical mask. For aCompCor, six components were calculated within the intersection of the subcortical mask and the union of CSF and WM masks calculated in T1w space, after their projection to the native space of each functional run. Frame-wise displacement (Power et al., 2014) was calculated for each functional run using the implementation of Nipype. Motion, as measured by mean FD for each participant, did not differ significantly between pre- (*M* = 0.14 mm, *SD* = 0.05) and post-learning scans (*M* = 0.14 mm, *SD* = 0.04), *t*_(51)_ = 0.11, *p* = 0.91, Cohen's *d* = 0.02. Thus, any observed changes in functional connectivity from pre-learning to post-learning scans cannot be attributed to differences in data quality or head motion.

#### 2.4.3 Preprocessing in CONN toolbox

Following FMRIPREP, functional data were imported into CONN Toolbox (RRID:SCR_009550; Whitfield-Gabrieli and Nieto-Castanon, 2012) and then smoothed, filtered and denoised prior to connectivity analyses. Smoothing used spatial convolution with a Gaussian kernel of 5 mm full width half maximum (FWHM). Denoising used a standard denoising pipeline (Nieto-Castanon, 2020) including the regression of potential confounding effects characterized by white matter timeseries (5 CompCor noise components), CSF timeseries (5 CompCor noise components), motion parameters and their first order derivatives (12 factors) (Friston et al., 1996), outlier scans (below 52 factors) (Nieto-Castanon, 2022), session effects and their first order derivatives (12 factors), and linear trends (2 factors) within each functional run. Volumes with a framewise displacement of >0.5 mm were scrubbed. No participants were excluded for head motion. We followed by filtering of the BOLD timeseries (Hallquist et al., 2013) between 0.01 and 0.0625 Hz to focus on background connectivity. The low-pass filter was set to 0.0625 Hz (16 s period) to reliably remove the 12 s stimulus frequency of the passive exposure tasks (Frank et al., 2019; Frank and Zeithamova, 2023; Tambini et al., 2017). This approach has been shown to produce comparable connectivity patterns and individual differences in connectivity values in slow event-related designs as the alternative approach, modeling out task-related activation using FIR and analyzing residuals, at a fraction of computational cost (Frank and Zeithamova, 2023). After band-pass filtering the data, CompCor noise components within white matter and CSF were estimated by computing the average BOLD signal as well as the largest principal components orthogonal to the BOLD average, motion parameters, and outlier scans within each subject's eroded segmentation masks. These estimates were used in subsequent connectivity analyses as nuisance regressors.

### 2.5 FMRI statistical analyses

#### 2.5.1 Overview

Analyses were conducted using CONN Toolbox and custom MATLAB scripts. The main goal of the study was to test the notion that the hippocampus supports both memory specificity and generalization but may be doing so through interaction with distinct cortical regions. To test this idea, we focused on background functional connectivity, a measure of low-frequency signal synchrony between regions after task-based fluctuations are removed. This measure can provide information about regional interactions, as well as how regional interactions are affected by different cognitive demands, or how they change in response to learning. Importantly, background functional connectivity reflects coordinated low-frequency signal fluctuations between regions and does not provide information about the direction of influence or causal interactions. Accordingly, associations between connectivity change and behavior are interpreted as correlational relationships rather than evidence of specific mechanisms.

Here, we measured hippocampal connectivity overall, how hippocampal interactions change following learning, and how connectivity changes predict subsequent memory for specific details (recognition) and the ability to generalize (categorization). Anterior and posterior hippocampus were analyzed separately, given their distinctive connectivity profiles (Tang et al., 2020) and differential implication in memory specificity vs. generalization processes (Frank et al., 2019; Poppenk et al., 2013). To complement the hippocampal analysis, we also performed an exploratory whole-brain segmentation ROI-to-ROI analysis to determine whole-brain connectivity changes that predict recognition and categorization.

#### 2.5.2 Seed-based hippocampal connectivity analysis

The primary analysis was a seed-based hippocampal connectivity analysis conducted in the CONN Toolbox. Because anterior and posterior hippocampus are thought to exhibit distinct connectivity profiles, we treated these regions as separate ROIs. Anterior and posterior hippocampus masks were defined at the group level after normalizing to MNI template. Masks were generated by segmenting the hippocampus along its longitudinal axis. We used a canonical boundary definition of Y = −21, as proposed by Poppenk et al. (2013). However, defining the ROI masks in this manner may introduce some spatial imprecision due to normalization and smoothing effects and the relatively coarse functional resolution. Thus, the two slices near the proposed boundary of Y = −21 (Poppenk et al., 2013) were discarded as potentially borderline, offering clearer separation. The posterior hippocampus ROI began at Y = −23, and the anterior hippocampus ROI began at Y = −19.

Two first-level seed-based connectivity maps were estimated for each participant, characterizing the spatial pattern of functional connectivity with the anterior hippocampus and posterior hippocampus respectively. Three second-level group analyses were performed using a General Linear Model, separately for each voxel (GLM, Nieto-Castanon, 2020). Voxel-level hypotheses were evaluated using multivariate parametric statistics with random-effects across subjects and sample covariance estimation across multiple measurements. Inferences were performed at the level of individual clusters (groups of contiguous voxels). Cluster-level inferences were based on parametric statistics from Gaussian Random Field theory (Nieto-Castanon, 2020; Worsley et al., 1996). Results were thresholded using a combination of a cluster-forming voxel-level threshold (that differed across analyses), and a familywise corrected p-FDR < 0.05 cluster-size threshold (Chumbley et al., 2010).

First, we aimed to replicate distinct anterior and posterior hippocampal connectivity profiles described previously in the current sample by directly contrasting anterior and posterior hippocampus connectivity across all runs (Tang et al., 2020). Because of prior work indicating robustly different connectivity profiles, we used stringent voxel and cluster thresholds (voxel-wise threshold of *p* < 0.001, cluster p-FDR < 0.05). To anticipate, we replicated distinct connectivity profiles for the two hippocampal ROIs, proceeding with the remaining analyses separately for anterior and posterior hippocampus. Second, we examined overall learning-driven hippocampal connectivity changes by contrasting connectivity patterns pre-learning and post-learning. These may reflect several experience-related changes, including learning-related memory reorganization, stimulus familiarity or perceptual fluency, consolidation of recently acquired motor-response associations, awake reactivation, etc. While not possible to attribute specific changes to specific processes, characterization of these learning related changes may still be informative as visual output remained constant. Because the hippocampal connectivity changes related to learning would be expected to be subtle compared to the overall connectivity patterns that are stable across time (Gratton et al., 2018), we used a more liberal voxel-wise threshold of *p* < 0.01 while retaining overall FDR at *p* < 0.05 at the level of clusters. Finally, we tested how hippocampal connection changes from pre-learning to post-learning related to subsequent recognition and categorization performance using multiple regression, with recognition and categorization scores as predictors and hippocampal connectivity change as an outcome. This approach allowed us to assess the brain-behavior relationship simultaneously for both kinds of memory behaviors, exploring the variance unique to each memory process. Because standard voxel-wise thresholds would require unrealistic brain-behavior correlation values, we set the voxel-wise threshold *p* < 0.03, which corresponds to a more realistic brain-behavior correlation of |r| = 0.3 with our sample size. Cluster threshold value was set at standard FDR-corrected *p* < 0.05.

#### 2.5.3 Exploratory cortico-cortical network connectivity analysis

To complement the seed-based hippocampal connectivity analysis and explore cortical contributions to face recognition and categorization abilities, we also performed an exploratory whole-brain ROI-to-ROI and network connectivity analysis. We used a cortical parcellation atlas provided by Schaefer et al. (2018), parcellating the brain into 100 distinct ROIs, with each ROI mapped to one of seven brain networks: default, somatomotor, dorsal attention, salience/ventral attention, visual, limbic, and control.

Using these ROIs, two 100 × 100 functional connectivity matrices (one pre-learning, one post-learning) were generated in CONN Toolbox for each subject by calculating the Pearson correlation coefficient between the time series of all ROI pairs. These matrices were used for later group analyses conducted using custom scripts in MATLAB. Additionally, we examined network-level functional connectivity by collapsing across the regions belonging to each network to further investigate how the network interactions change in response to the category learning task.

## 3 Results

### 3.1 Behavioral results

#### 3.1.1 Category learning performance

**Figure 3:**
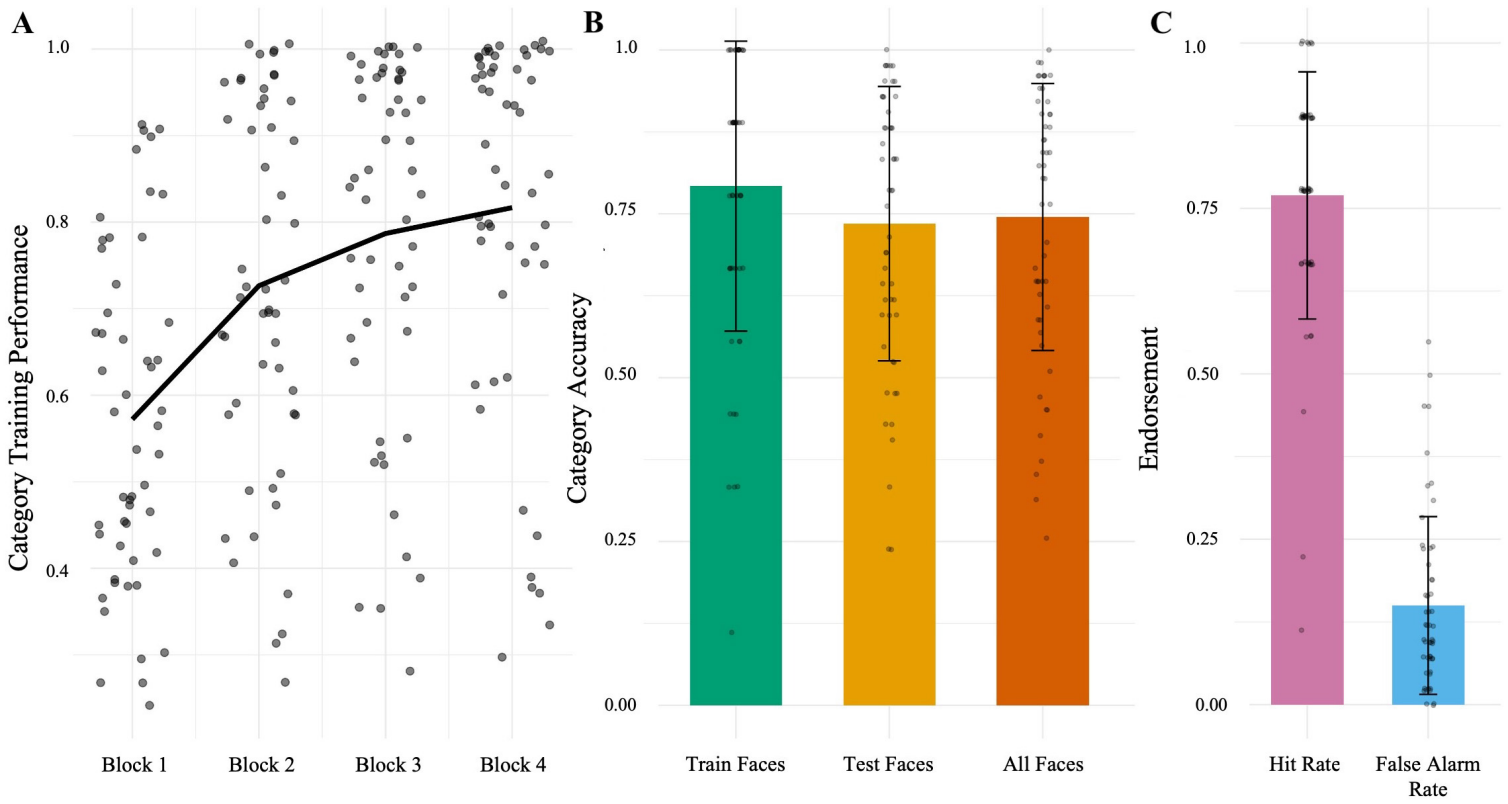
Behavioral performance. **(A)** Category training performance. Participants scored significantly above chance (33%) in the category learning task, with performance steadily increasing across training blocks. **(B)** Category generalization performance. Participants performed significantly above chance (33%) on the category generalization task for both old and new faces. **(C)** Recognition performance. Participants performed significantly above chance (0%) on the recognition task.

We first examined categorization accuracy from the category learning task to assess how well participants were able to learn the family categories. Participants successfully learned the categories and performed significantly above chance (33%) on this task (Figure 3A, mean accuracy = 0.73 ± 0.19, *t*_(51)_ = 15.1, *p* < 0.001).

#### 3.1.2 Categorization test performance

During the final categorization test, participants correctly categorized 74% (SD = 20%) of test faces (Figure 3B), which is well above chance (33.3%; *t*_(51)_ = 14.3, *p* < 0.001). The successful categorization of both training and new faces suggests that category information was acquired during category learning and successfully generalized to the new faces. Nevertheless, participants differed with respect to how well they were able to learn the categories (range 26%−100% correct, split-half reliability with Spearman-Brown correction *r* = 0.86), allowing us to look for neural predictors of individual differences.

#### 3.1.3 Recognition performance

Hit rates and false alarm rates in the face recognition task are presented in Figure 3C. The average corrected hit rate for participants was 62% (SD = 21%) which was well above chance (chance corrected hit rate = zero, *t*_(51)_ = 20.9, *p* < 0.001). Thus, participants were able to differentiate training faces from similar new faces. Furthermore, we observed large individual differences in recognition performance (corrected hit rate range 10%−95%, split-half reliability with Spearman-Brown correction *r* = 0.75).

#### 3.1.4 Dissociation of recognition and category generalization performance

To test to what degree the recognition and category generalization tasks were measuring distinct behaviors, we tested their correlation. We found that the recognition and category generalization tasks were not correlated with each other in the current paradigm (*r* = 0.05, *p* = 0.72), in line with our prior work (Bowman et al., 2022). Thus, although neither categorization nor recognition would be expected to be a process-pure measure, the high reliability of each measure and the lack of correlation between them indicates that each task is measuring a different cognitive process.

### 3.2 Hippocampal connectivity

#### 3.2.1 Differences in anterior and posterior hippocampal connectivity

**Figure 4:**
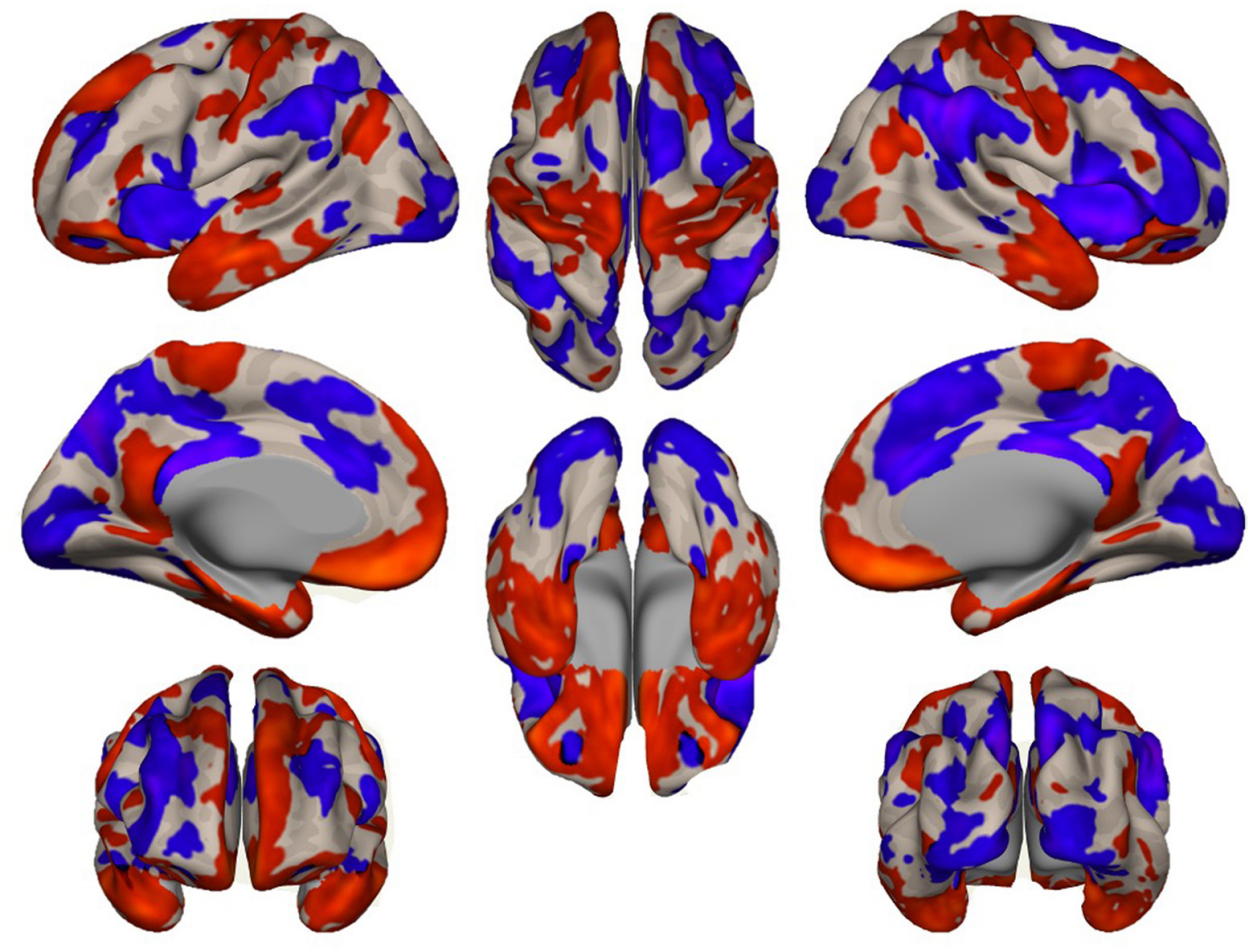
Difference in functional connectivity between anterior and posterior hippocampus across all functional runs (pre-learning and post-learning). Red: higher functional connectivity with anterior than posterior hippocampus; blue: higher functional connectivity with posterior than anterior hippocampus; voxel *p* < 0.001, cluster *p* < 0.05.

Before treating anterior and posterior hippocampus as separate regions, we first contrasted anterior and posterior hippocampal connectivity across the average of all four task runs (two pre-learning and two post-learning) to confirm previous findings that they form distinct connections across the brain (Figure 4). Anterior and posterior hippocampus had significant differences in connectivity that were largely consistent with previous findings (Tang et al., 2020). As expected, regions of the middle temporal gyrus and ventromedial PFC showed greater connectivity to anterior hippocampus, and lateral prefrontal and lateral parietal cortices were more strongly connected with the posterior hippocampus.

#### 3.2.2 Changes in anterior and posterior hippocampal connectivity after category learning

**Figure 5:**
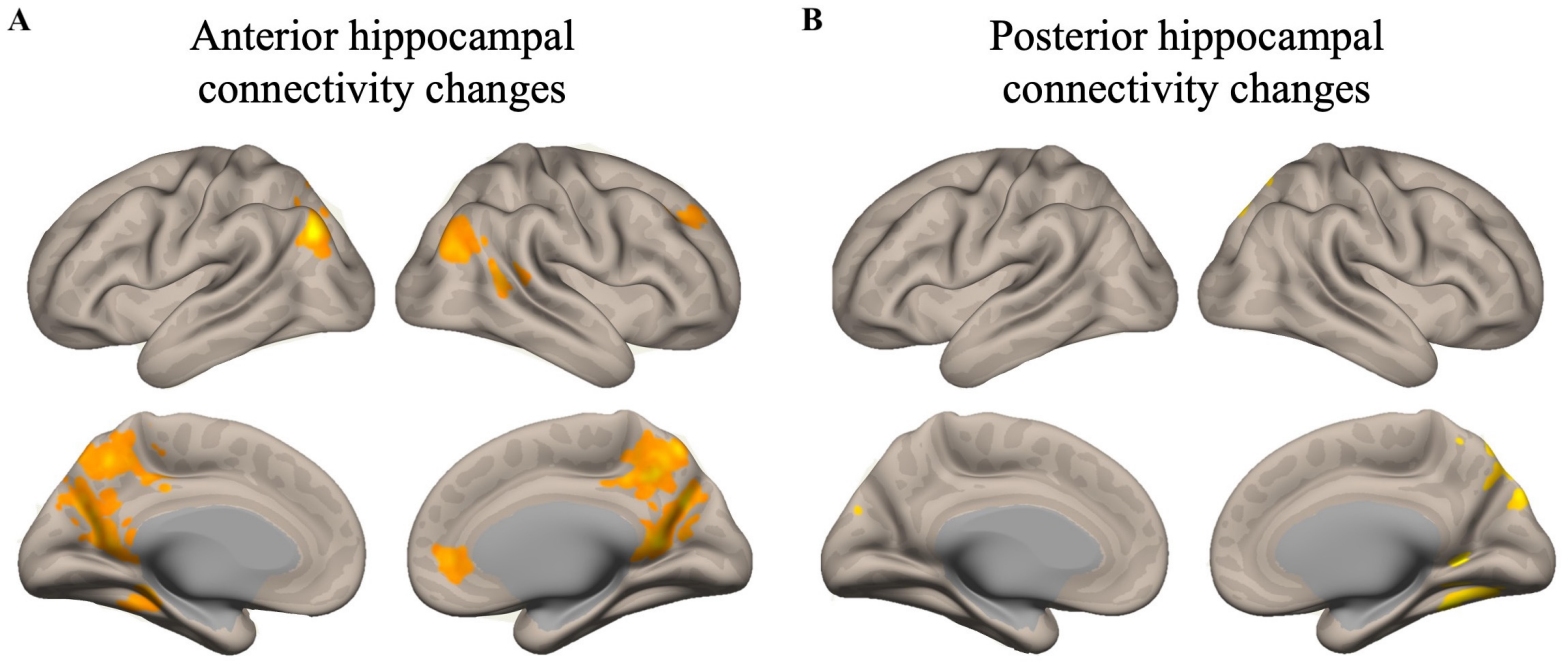
Hippocampal connectivity changes from pre-category learning to post-category learning. **(A)** Anterior hippocampal connectivity changes (voxel *p* < 0.01, cluster *p* < 0.05). Anterior hippocampus increased in connectivity with bilateral superior lateral occipital cortex, right frontal pole, right angular gyrus, right middle temporal gyrus, left posterior fusiform cortex, precuneus, and posterior cingulate cortex. **(B)** Posterior hippocampal connectivity changes (voxel *p* < 0.01, cluster *p* < 0.05). Posterior hippocampus increased in connectivity with right fusiform cortex, right cuneal cortex, precuneus, and posterior cingulate cortex.

Next, we examined how the anterior and posterior hippocampal connections changed after category learning. For each region, we contrasted its connectivity during pre-category learning passive-viewing and post-category learning passive-viewing (post > pre) to establish how connectivity between the hippocampus and neocortex changed as a function of the learning task. Anterior hippocampus showed increased connectivity with bilateral superior lateral occipital cortex as well as several regions of the default mode network, including medial PFC, precuneus, right angular gyrus, and right middle temporal gyrus (Figure 5A). Posterior hippocampal connectivity changes were less widespread, and included primarily increased connectivity with fusiform cortex, precuneus and cuneus (Figure 5B).

#### 3.2.3 Changes in anterior and posterior hippocampal connectivity predicting recognition and categorization performance

We next addressed whether changes in anterior and posterior hippocampal connectivity predicted performance on later assessments of recognition and categorization abilities. As standard voxel-level thresholds would require unrealistically large brain-behavior correlations, we used a more liberal voxel level threshold of *p* < 0.03, corresponding with our sample size to a more realistic brain-behavior correlation of *r* = 0.3. Nevertheless, we then used standard FDR *p* < 0.05 cluster-corrected threshold to ensure robustness.

We hypothesized that hippocampal connections with medial prefrontal and other generalization regions would predict categorization, while hippocampal connections with regions implicated in memory specificity and differentiating similar events, such as ventrolateral PFC, would predict recognition. Furthermore, we hypothesized that anterior hippocampal connectivity changes would be predictive of categorization while posterior hippocampal connectivity changes would be predictive of recognition. To test these hypotheses, recognition and categorization performance were modeled simultaneously using multiple regression, allowing identification of connectivity effects uniquely associated with each ability while controlling for their shared variance.

**Figure 6:**
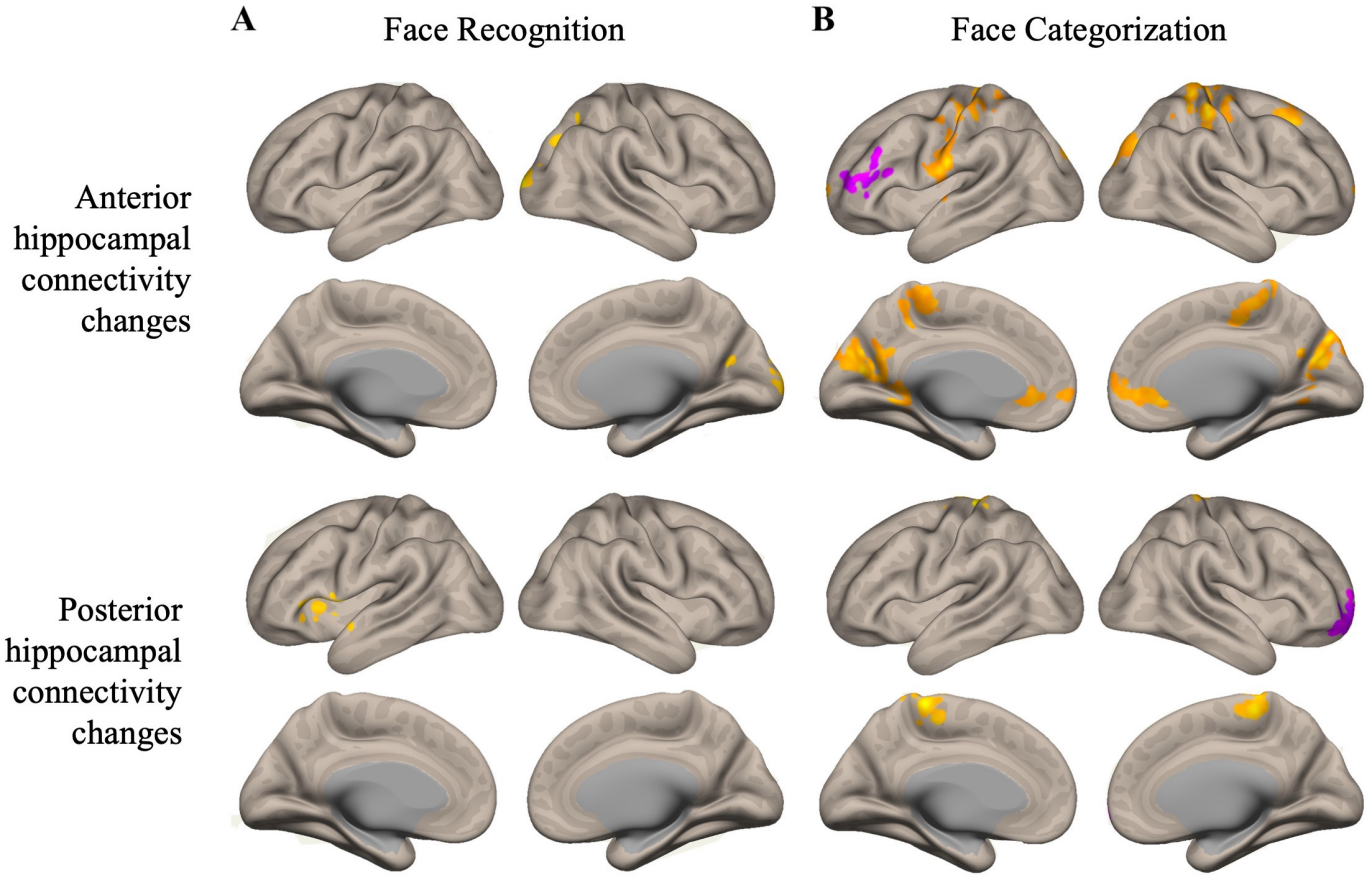
Hippocampal connectivity changes predicting memory performance. Multiple regression was used to identify connections associated with one memory process while controlling for the other memory process. **(A)** Anterior and posterior hippocampal connectivity change predicting face recognition performance. **(B)** Anterior and posterior hippocampal connectivity change predicting face categorization performance. Voxel level threshold: correlation with behavior *r* = 0.3 (*p* < 0.03, cluster corrected at *p* < 0.05).

The results partially but not fully aligned with our hypotheses. First, consistent with our hypothesis, we found that recognition and categorization abilities were tracked by predominantly distinct hippocampal connections. Recognition ability (Figure 6A) was predicted by increased connectivity between anterior hippocampus and right early visual and dorsal visual regions, as well as posterior hippocampus and left ventrolateral PFC (inferior frontal gyrus). Categorization ability (Figure 6B) was predicted by increased connectivity between anterior hippocampus and ventromedial PFC, somatomotor cortex, and early visual cortex, as well as decreased connectivity between posterior hippocampus and a superior frontal/frontal pole region. Additionally, categorization ability was predicted by increased connectivity between both anterior and posterior hippocampus and paracentral lobule. The anterior hippocampus-medial prefrontal connectivity predicting categorization and posterior hippocampus-lateral prefrontal connectivity predicting recognition align with the regions' hypothesized roles in memory generalization and memory specificity, respectively.

### 3.3 Exploratory analysis of whole-brain cortico-cortical connectivity

#### 3.3.1 Whole-brain cortico-cortical connectivity changes after category learning

**Figure 7:**
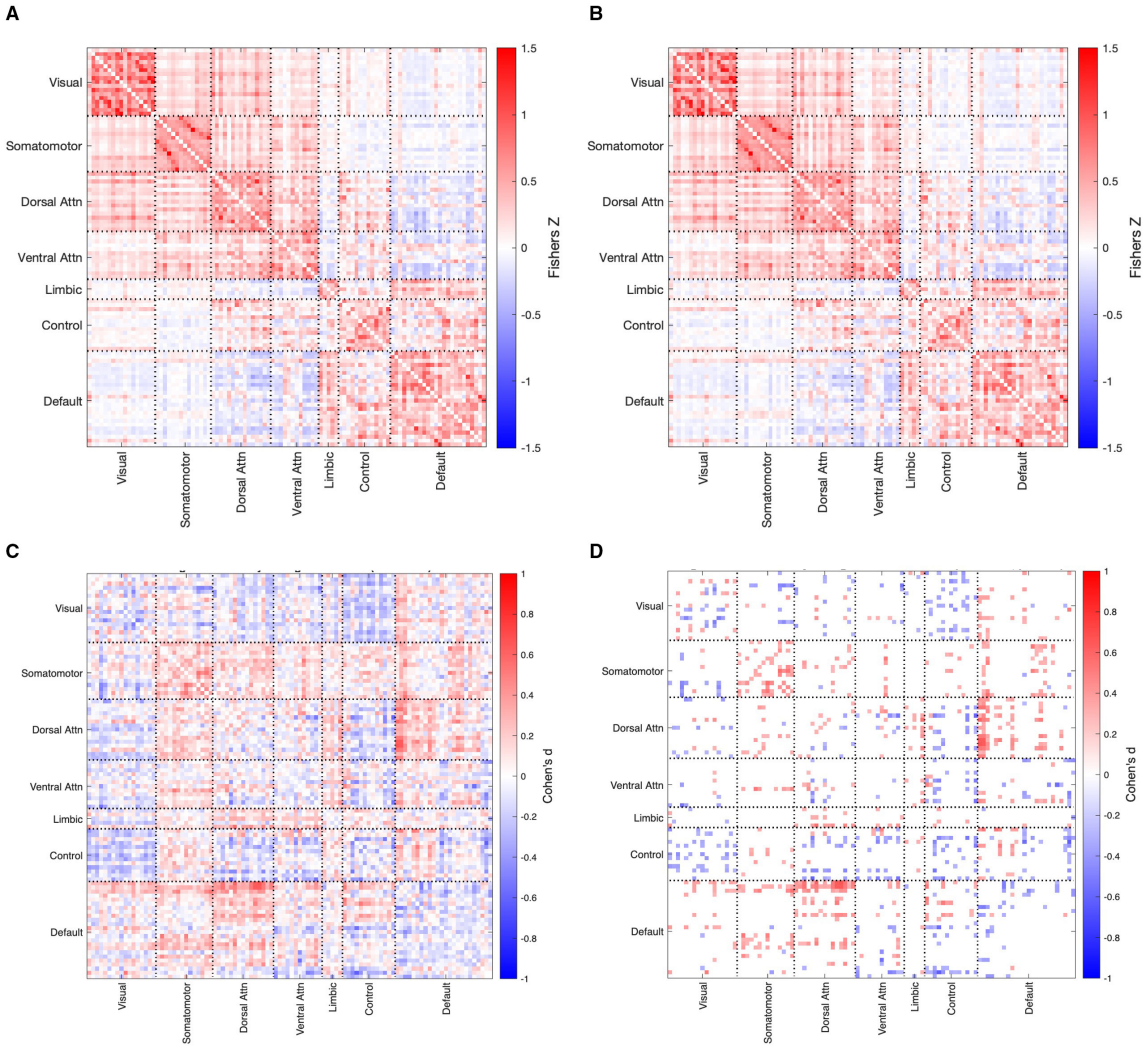
Whole-brain connectivity before and after category learning. **(A)** ROI-level pre-category learning connectivity. **(B)** ROI-level post-category learning connectivity. **(C)** Effect size of ROI-level connectivity changes after learning (Cohen's *d*). **(D)** ROI-level significant connectivity changes after learning.

As the hippocampus is not the only region supporting learning and memory, we further explored the extent to which category learning impacts cortico-cortical connectivity, using a whole-brain ROI-to-ROI connectivity analysis. Importantly, this analysis was designed to complement the hippocampal seed-based analyses. Whereas, the seed-based approach tested our specific hypotheses about how hippocampal connectivity changes relate to recognition and categorization performance, this whole-brain cortico-cortical connectivity analysis provided an unbiased survey of learning-related connectivity changes across the entire cortex. For this analysis we used the Schaefer et al. (2018) atlas, segmenting whole cortex in 100 parcels, attributed to 7 networks. This provides means to explore all of the cortex in a data-driven way while avoiding the computational and interpretational challenges of voxel-level analyses. Three 100 × 100 connectivity matrices (Figures 7A–C) were used to visualize the connectivity strength between of each pair of regions before category learning, after category learning, and how the connectivity changed after category learning. Each unique connection is visualized both on upper diagonal and lower diagonal. Individual connections in the ROI-to-ROI analyses were tested at an uncorrected threshold (*p* < 0.05). Rather than interpreting individual connections, we evaluated statistical significance at the pattern level by testing whether the number of significant connections exceeded chance expectations using binomial tests. Given this exploratory analytical framework, we focus our interpretation on network-level summaries and convergent patterns across multiple connections rather than on any single ROI-to-ROI connection.

As expected, both before and after category learning, the cortex displayed a coherent network structure, with regions being more connected to one another when they belonged to the same network (Figures 7A, B). Of main interest was the change in connectivity following learning. Figure 7C shows the raw connectivity changes, Figure 7D visualizes all connections that changed exceeding an uncorrected threshold of *p* < 0.05 for descriptive purposes; all statistical inference is evaluated at the pattern level. As it would not be feasible to correct for multiple comparisons at the level of individual connections (4,950 unique connections between 100 parcels, excluding self-connections), we evaluated for significance at the whole pattern level. In total, we observed about 11%, or 535 out of 4,950 unique connections to change after learning, which is significantly more connections than 5% expected just by chance (probability of 535 or more false positives given 0.05 probability of each positive, *p* < 0.001, binomial test). Thus, the pattern of cortico-cortical connections changed in response to the learning experience.

**Figure 8:**
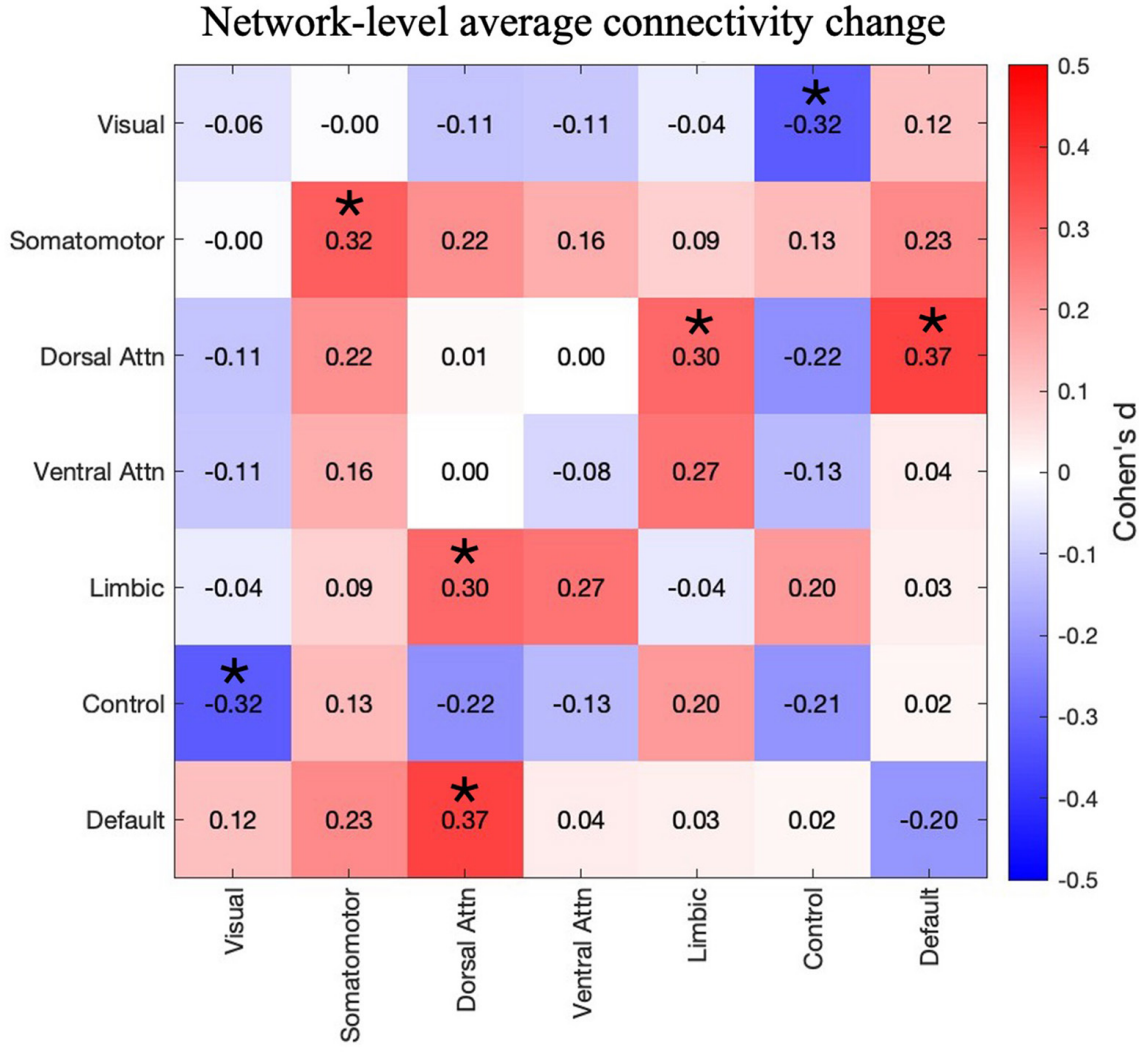
Network-level connectivity changes after category learning. Numbers show the effect size of connectivity change (Cohen's *d*). Stars indicate significant changes in response to learning at uncorrected *p* < 0.05.

Looking at the pattern of connections in Figure 7D, we were interested whether these changes were driven by any particular networks. We collapsed the ROI-to-ROI connectivity matrices by averaging all connections that belong to the same pair of Schafer-defined networks and then subtracted pre-learning and post-learning matrices in each participant. This provided one average connectivity change value for each pair of networks for each person. The average network-to-network connectivity changes from pre-category learning to post-category learning are visualized in Figure 8. Evaluated at the network level, we replicated the ROI-level finding that the overall connectivity pattern changed reliably, with 4 out of 28 unique connections (14%) passing uncorrected threshold of 0.05 (*p* = 0.049, binomial test). Somatomotor, limbic–dorsal attention, and default–dorsal attention network connectivity all significantly increased, and visual–control network connectivity significantly decreased from pre- to post-category learning. Thus, while individual connections cannot be interpreted in isolation, the results suggest that learning-related reconfiguration may be non-uniform across networks.

#### 3.3.2 Exploratory whole-brain cortico-cortical connectivity predicting behavior

**Figure 9:**
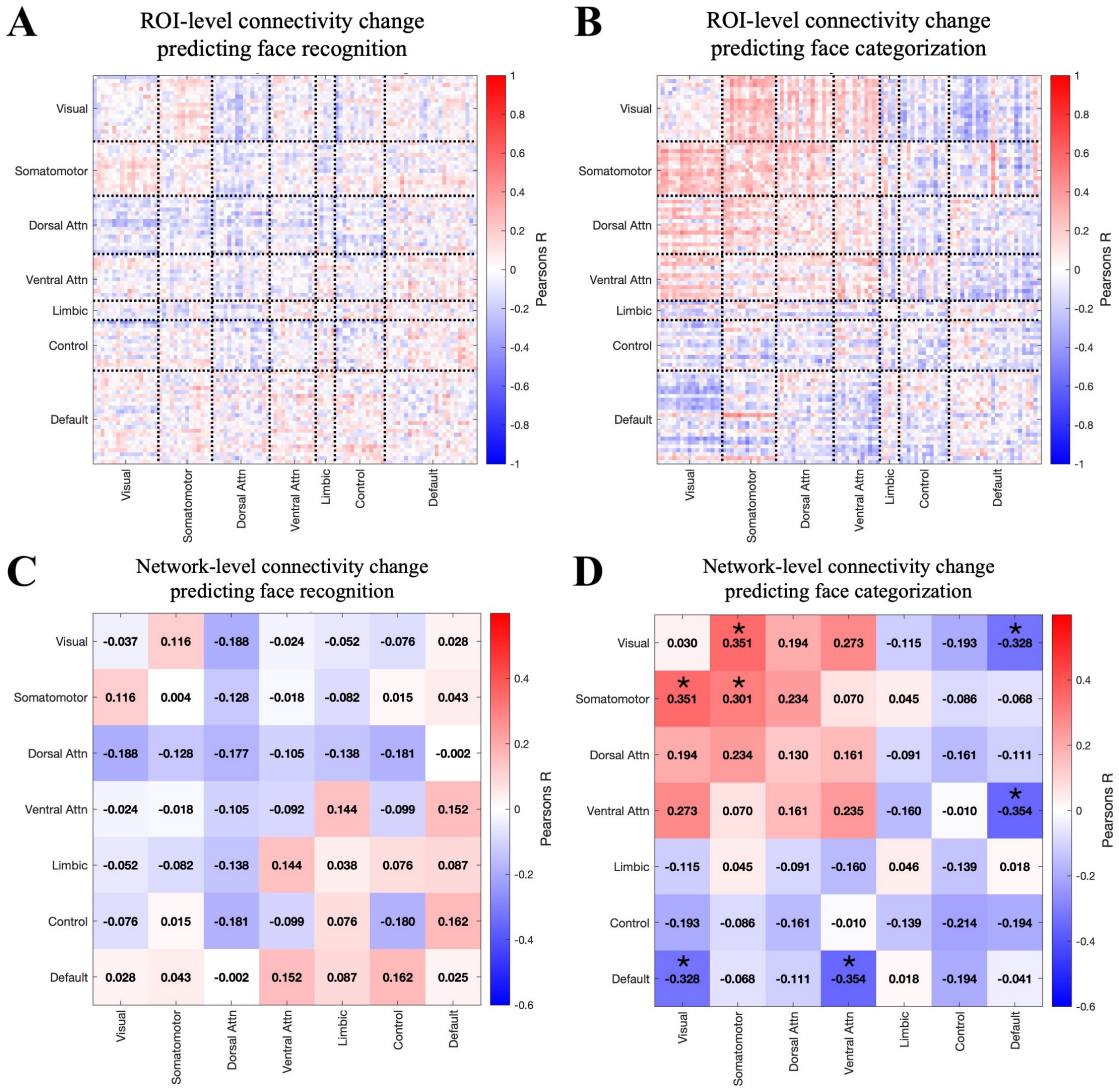
Connectivity change predicting memory performance. **(A)** Across-participant correlations between ROI-level connectivity change and recognition performance. **(B)** Across-participant ROI-level connectivity change and categorization performance. **(C)** Across-participant correlations between network-level connectivity change and recognition performance. **(D)** Across-participant correlations between network-level connectivity change and categorization ability. Stars denote correlation values significant at uncorrected *p* < 0.05.

Finally, we examined how changes in ROI and network connectivity after category learning predicted later performance on recognition and categorization measures. First, using the 100 × 100 ROI connectivity matrix, we correlated participants' connectivity values with their performance on the recognition and categorization assessments. This generated a correlation matrix for the relationship between the change in ROI connectivity and recognition and categorization performance. Figures 9A, B show the correlational values for all pairs of ROIs, with each unique correlation shown on both upper and lower diagonals.

To more formally test whether cortical connectivity changes predicted behavior, we ran a multiple linear regression for each connection across participants, with recognition and categorization as statistical predictors and connectivity strength as an outcome. The resulting beta and *p-value* matrices were nearly identical to raw correlation matrices, which is expected with uncorrelated predictors. The number of connections that predicted recognition performance (at uncorrected *p* < 0.05) was 144 out of 4,950, or about 3%, which can be expected just by chance (binomial test *p* = 1.0). For categorization, we found 572 ROI-ROI connections, or about 11.6% of all connections, that exceeded an uncorrected threshold of *p* < 0.05 in their association with categorization performance. This significantly surpasses the number of connections expected by chance (*p* < 0.001, binomial test). Thus, cortico-cortical connectivity changes were reliably linked to individual differences in subsequent categorization performance.

After determining that face categorization was significantly predicted by changes in cortico-cortical connectivity, we then examined whether this finding was driven by specific networks. We again collapsed connectivity values across parcels belonging to the same pairs of networks, and correlated participants' network connectivity changes with their performance on the recognition and categorization assessments. Figures 9C, D show the correlation matrices between connectivity changes and performance for all pairs of networks. Consistent with the ROI-ROI results, we observed no significant correlations between network connectivity change and face recognition performance, even at an uncorrected threshold of *p* < 0.05 (Figure 9C). Four network-network connections were correlated with face categorization at uncorrected *p* < 0.05: increased connectivity within somatomotor network and between visual–somatomotor network and decreased connectivity between visual–default and ventral attention–default networks (Figure 9D). This surpasses the number of significant connections expected by chance (*p* = 0.049, binomial test). In combination with the reliable ROI-ROI results, these exploratory results emphasize convergent network-level patterns: behavioral performance was associated with distributed changes that, when summarized on a network level, highlighted a small number of network-pair interaction changes (Figures 9B, D).

## 4 Discussion

The present study investigated changes in hippocampal–cortical connectivity following a learning experience and examined how these changes relate to individual differences in memory specificity and memory generalization within the same learning context. Using a category learning task that provided participants the opportunity to form both detailed and generalized representations, we found widespread task-induced connectivity change in the anterior hippocampus with more limited connectivity changes in the posterior hippocampus. Behavioral data indicated that participants formed both specific representations of individual faces and generalizable category knowledge. These appeared to rely on distinct memory processes as recognition and categorization success were uncorrelated. Linking individual differences in behavior to hippocampal connectivity changes, we found distinct sets of hippocampal connections predicting face recognition and face categorization performance that partially aligned with hypothesized specificity vs. generalization dissociations within the hippocampus and cortex. The results are consistent with the idea that the hippocampus supports both memory specificity and generalization through interactions with distinct cortical regions.

Recent work highlights that the hippocampus does not just support the ability to remember differentiating details (Eichenbaum, 2000; Scoville and Milner, 1957; Tulving and Markowitsch, 1998), but also supports our ability to apply previously learned information to new experiences (Preston et al., 2004; Shohamy and Wagner, 2008; Zeithamova et al., 2008, 2012). Our results showing hippocampal connectivity changes predicting both recognition and categorization lend further support to that notion. Consistent with the implicated roles of distinct prefrontal regions in memory specificity vs. generalization, we found hippocampal-ventrolateral PFC connections to predict recognition while hippocampal-ventromedial PFC connections predict categorization. These results replicate prior connectivity findings that link hippocampal-ventrolateral connectivity to memory for specific details and resistance to interference between similar memories (Badre and Wagner, 2005; Bowman and Dennis, 2016; Kuhl et al., 2007) and hippocampal-ventromedial connectivity to memory integration, generalization and schema-related memory processing (Bowman and Zeithamova, 2018; Schlichting et al., 2015; Van Kesteren et al., 2013; Zeithamova et al., 2012). Furthermore, we replicate and extend prior findings showing increased hippocampal connectivity with visual stimulus processing regions following encoding that predict subsequent memory (Liu et al., 2018; Tambini et al., 2010; Tompary et al., 2015). Newly, we are demonstrating these links in a single paradigm, testing memory for differentiating details and integration across experiences in response to the same learning episode, in the same group of participants.

Within the hippocampus, we further tested how hippocampal connectivity and its relationship to memory behaviors vary along its anterior-posterior axis. We found relatively widespread connectivity increases between anterior hippocampus and several visual and default-mode network regions following category learning, while posterior hippocampal connectivity changes were more limited and primarily involved visual cortices and small portions of precuneus. We hypothesized that anterior hippocampal connections with putative generalization regions would be associated with categorization performance while posterior hippocampal connections with putative specificity regions would be associated with recognition. These predictions were based on the hypothesis that anterior hippocampus preferentially supports generalization, whereas posterior hippocampus is more critical for forming specific representations of information (Bowman and Zeithamova, 2018; Brunec et al., 2018; van Geen et al., 2025; Komorowski et al., 2013; Kumaran et al., 2009; Poppenk et al., 2013; Zeithamova et al., 2008). The results partially but not fully aligned with this prediction. Consistent with our prediction, we found that recognition ability was predicted by increased connectivity between posterior hippocampus and left ventrolateral PFC while generalization ability was predicted by increased connectivity between anterior hippocampus and ventromedial PFC. These findings align with the proposed gradient in representational scale along the hippocampal axis (Brunec et al., 2018; Poppenk et al., 2013) and provide novel evidence that connectivity changes following learning relate to these functions.

Nevertheless, there were additional connections not predicted a priori and not clearly aligning with the hypothesized anterior/posterior dissociations. For example, both anterior and posterior hippocampal connections with paracentral lobule were predictive of categorization, likely reflecting the category learning procedure where participants indicated category membership via a button press and then received feedback. Interestingly, the hippocampal-sensorimotor cortex connectivity changes were observed even though participants were not actively responding during the functional MRI scan itself. Furthermore, both recognition and categorization performance was predicted by anterior hippocampal connections with early visual cortex. Others have implicated a modulation of anterior hippocampus—visual cortex coupling in visual memory precision (Xie et al., 2025), and both anterior and posterior hippocampal connectivity track memory quality in a task focused on memory precision with no difference between the anterior and posterior subregions (Cooper and Ritchey, 2019; Xie et al., 2023). One possibility why the anterior hippocampal-visual cortex connectivity appears associated with both specificity and generalization could be due to schematic information from the hippocampus facilitating the retrieval of both coarse-grain and detailed visual representations. While we cannot attribute the connection to a specific underlying mechanism based on current data, these findings add nuance to theories emphasizing division of labor along the anterior/posterior hippocampal axis.

Another reason for the nuanced pattern may be that computations within hippocampal subfields may interact with the representational gradient along the long axis. Theoretical accounts propose that distinct subfields support complementary memory functions: CA3 and dentate gyrus subfields have been implicated in encoding of specific events and pattern separation of similar experiences, while CA1 is thought to compare incoming information with stored representations and integrate information across experiences (Rolls, 2013; Schapiro et al., 2017; Sučević and Schapiro, 2023; Treves and Rolls, 1994). Empirically, high-resolution fMRI studies have shown that DG/CA3 pattern-separation circuitry is preferentially engaged during mnemonic discrimination, whereas CA1 is more involved in generalization across overlapping experiences (Bakker et al., 2008; Barron et al., 2020; Lacy et al., 2011; Schlichting et al., 2014; Yassa and Stark, 2011). Our findings of distinct connectivity patterns may reflect not only anterior-posterior organization, but also differential engagement of subfield circuits, which are distributed along the long axis non-uniformly (González-Arnay et al., 2024; Malykhin et al., 2010; Poppenk et al., 2013). Taken together, these findings suggest that the representational gradient along the long-axis and subfield-specific computations are likely interacting influences on hippocampal-cortical dynamics. Future work incorporating analyses targeting specific hippocampal subfields will be necessary to fully characterize how these different organizational levels interact to support adaptive memory function.

Beyond hippocampal–cortical connections, we also explored cortico-cortical connections without specific predictions. Because these analyses relied on pattern-level binomial tests and uncorrected connection-level thresholds, they were intended to provide an initial descriptive characterization of large-scale network changes rather than evidence for specific mechanistic pathways. Within this exploratory framework, we found significant changes in cortico-cortical connections in response to learning, that were primarily driven by increased connectivity in dorsal attentional, default, limbic, and somatosensory network, and decreased connectivity between control and visual networks. Furthermore, cortico-cortical connectivity changes were predictive of individual differences in subsequent categorization success, which appeared driven by decreased connectivity between the default mode network and visual and ventral attentional networks, and increased visuo-somatomotor and within-somatomotor connectivity. As with the hippocampal findings, the increase in connectivity within somatomotor network regions and between somatomotor and visual network regions may be reflective of the task demands, learning to associate groups of visual stimuli with corresponding button-presses. Additionally, our findings showing default mode network connectivity predicting categorization extend previous findings linking the default mode network connectivity to memory performance (Kaefer et al., 2022; Sestieri et al., 2011; Wahlheim et al., 2022; Xu et al., 2025). While most studies have focused on intra-default connectivity (Dunn et al., 2014; Ward et al., 2015; Xu et al., 2025) showed default connectivity, including connectivity with sensory regions and salience network, to predict forgetting of word associations. Our findings suggest that the default mode network may also play a more active role in the elaborative process of memory generalization. Interestingly, better categorization was associated with learning-related *decrease* in default-visual and default-salience connectivity, perhaps reflecting that the formation of conceptual knowledge requires some abstraction away from specific stimulus details. Taken together, these exploratory findings indicate that learning-related reorganization may extend across large-scale cortical systems, raising the possibility that generalization depends in part on the integration of perceptual and conceptual networks. However, further work with more targeted whole-brain analyses will be needed to establish the robustness and mechanistic significance of these network-level effects.

Our findings also contribute to the ongoing debate about whether memory is a single faculty or is comprised of multiple interacting systems (Henson and Gagnepain, 2010; Knowlton and Squire, 1993; Nadel, 1992; Nosofsky et al., 2012; Sherman et al., 2024; Sherry and Schacter, 1987). Recent attention to hippocampal contributions to some forms of categorization may appear to support a single system view (Nosofsky et al., 2012; Nosofsky and Zaki, 2003), However, several features of our data, such as uncorrelated individual differences and the distinct connectivity patterns associated with recognition and categorization, are difficult to fully reconcile with single-system accounts that rely solely on graded representational differences. Rather, they provide support for the view that memory is not a monolithic process but instead reflects the coordinated operation of partially distinct systems where separable neural mechanisms are optimized for different memory demands (Schapiro et al., 2017; Sherman et al., 2024; Zeithamova and Bowman, 2020). By demonstrating that specificity- and generalization-related processes are supported by distinct hippocampal-cortical connections, our results reinforce the idea that different memory behaviors arise from the interaction of specialized systems, each contributing unique representational and computational properties to adaptive cognition.

While the current findings provide novel insights into hippocampal contributions to memory specificity and generalization, several limitations should be noted. First, our sample was restricted to young adults, and it remains to be determined whether these observed connectivity-behavior correlations extend across the lifespan. Mapping connectivity-behavior relationship in broader range of ages would be especially informative given known connectivity changes in aging (Chan et al., 2014; Deery et al., 2023; Varangis et al., 2019) and given that the ability to recognize and categorize information is affected differentially as we age (Bowman et al., 2019, 2022; Charles and Bowman, 2025; Mutter and Plumlee, 2014; Simon and Gluck, 2013). Second, we used a single gender (male) and single race (white) face stimuli as creating face-blends with mixed-gender or mixed-race faces would introduce preexisting categories into our planned category structure. However, given existing work on own-race and own-gender biases in memory effects (Herlitz and Lovén, 2013; Meissner and Brigham, 2001; Wright and Sladden, 2003), this may limit the generalizability of our findings. Additionally, faces are a unique stimulus type (Diamond and Carey, 1986; Hong Liu and Chaudhuri, 2003; Kanwisher and Yovel, 2006; Yin, 1969; Yue et al., 2006) and thus some of our findings may be unique to faces rather than stimulus-general. For example, we found that the interaction of the hippocampus and fusiform cortex predicted face recognition ability, which may be driven by the use of face stimuli (Geiger et al., 2016; Sliwinska et al., 2022; Zeineh et al., 2003). Additionally, while we found hippocampal connections predicting both recognition and categorization, whole-brain cortico-cortical analysis did not find any predictors of recognition beyond what would be expected just by chance. This may be driven by the explicit demands of a category learning task, as other studies using more traditional memory tasks found cortical connectivity predictors of memory for specific events (Kaefer et al., 2022; Sestieri et al., 2011; Wahlheim et al., 2022; Xu et al., 2025). It is also likely that the contrast of pre-post changes is suboptimal for capturing learning of individual faces, which would be expected to happen continuously, including during the passive viewing scans themselves. Finally, while our sample size is relatively large for an fMRI study, it is not fully powered for individual differences analysis, and thus any null effects should not be overinterpreted. Future studies using more diverse participant samples and stimulus sets while still employing paradigms that capture both recognition and categorization ability for the same experience are needed to establish the generalizability of these connectivity-behavior relationships.

In conclusion, the current study identified distinct patterns of hippocampal connectivity to be associated with individual differences in recognition and categorization, aligning with the idea that the hippocampus contributes to memory specificity and generalization through interactions with distinct cortical regions. The results further indicate that large-scale cortical networks also contribute to these processes. We found mixed evidence for functional dissociations for memory specificity and generalization along the long axis of the hippocampus and across cortex, with several but not all findings aligning with the hypothesized anterior/posterior division of labor. These findings advance our understanding of how the hippocampus and cortex cooperate to balance competing memory demands, a critical feature of adaptive memory. They also support the notion that memory is not a single faculty but is comprised of multiple complex systems. The interaction between the hippocampus and neocortex allows people to form multiple representations of the same experiences to support later memory and grants us a more expansive experience of the world.

## Data Availability

The datasets presented in this study can be found in online repositories. The names of the repository/repositories and accession number(s) can be found below: https://osf.io/5h8b7.
